# Impact of Short‐Term Insulin Pump Therapy on Cardiometabolic Index in Patients With Newly Diagnosed Type 2 Diabetes Mellitus

**DOI:** 10.1002/edm2.70192

**Published:** 2026-03-05

**Authors:** Xia Chen, Shuya Li, Zeren Chen, Dong Wang, Ling Yang, Xia Deng, Haoxiang Li

**Affiliations:** ^1^ Department of Endocrinology Affiliated Hospital of Jiangsu University Zhenjiang Jiangsu China

**Keywords:** cardiometabolic index, continuous subcutaneous insulin infusion, insulin resistance, newly diagnosed type 2 diabetes mellitus

## Abstract

**Objective:**

This study aims to investigate the changes in cardiometabolic index (CMI) levels in newly diagnosed type 2 diabetes mellitus (T2DM) patients before and after undergoing short‐term continuous subcutaneous insulin infusion (CSII) intensive treatment and analyse its correlation with insulin resistance.

**Methods:**

This study retrospectively collected data from 604 patients who were initially diagnosed with T2DM and received short‐term CSII treatment during their hospitalisation in the Endocrinology Department of the Affiliated Hospital of Jiangsu University. Clinical data, blood glucose, insulin, C‐peptide, blood biochemistry, and other indicators were collected before and after treatment, and CMI was calculated.

**Results:**

Short‐term CSII treatment significantly improved blood glucose levels, insulin resistance, and pancreatic β‐cell function in T2DM patients, while downregulating the CMI index (*p =* 0.002). Correlation analysis showed a positive correlation between CMI and HOMA‐IR (*p* < 0.001) and a negative correlation between CMI and HOMA‐β (*p* = 0.008). Stepwise linear regression model analysis revealed that CMI was independently associated with fasting C‐peptide (FCP) before treatment (*β* = 0.291, *p* < 0.001); after treatment, CMI was associated with FCP (*β* = 0.160, *p* < 0.001) and HOMA‐IR (*β* = 0.159, *p* < 0.001).

**Conclusion:**

After short‐term intensive CSII treatment, the CMI level in newly diagnosed T2DM patients significantly decreased, indicating that intensive CSII treatment may have certain significance in improving cardiometabolic function.

## Graphical abstract

The study reveals that CMI levels significantly decrease after short‐term CSII therapy in T2DM, with CMI positively correlated with insulin resistance, indicating the therapy's significance in improving patients' cardiometabolic function.

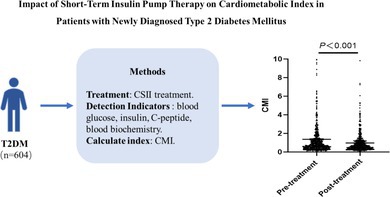

## 1 Introduction

Cardiometabolic Index (CMI), as an important indicator reflecting the state of myocardial energy metabolism, has gradually garnered attention in the assessment of insulin resistance and metabolic abnormalities [1, 2, 3]. By comprehensively considering the waist‐to‐height ratio (WHtR) and the ratio of triglyceride (TG) to high‐density lipoprotein cholesterol (HDL‐C), CMI can comprehensively reflect the impact of abdominal obesity and dyslipidemia on cardiovascular and metabolic health [2]. Compared with the non‐diabetic control group, the CMI in the type 2 diabetes mellitus (T2DM) group was significantly elevated, and CMI was negatively correlated with the early‐phase insulin secretion index [4]. Wu et al. found that in T2DM patients, CMI was positively correlated with the Homeostatic Model Assessment of Insulin Resistance (HOMA‐IR). Logistic regression analysis revealed that CMI was an independent influencing factor for insulin resistance (male: OR = 1.30, 95% CI = 1.15–1.47, female: OR = 1.62, 95% CI = 1.32–1.99) [5]. Continuous subcutaneous insulin infusion (CSII) therapy is a treatment modality that employs a programmable continuous infusion system to provide a constant basal rate of insulin over 24 h, with additional bolus doses administered during meals or when blood glucose levels rise [6, 7]. For T2DM patients, compared with multiple daily insulin injections, the advantage of CSII lies in its ability to more flexibly adjust the basal insulin infusion rate, thereby better controlling blood glucose variability, reducing the risk of nocturnal hypoglycemia, and improving overall glycemic stability [8, 9]. Studies have shown that in T2DM patients receiving CSII therapy, the level of glycated haemoglobin (HbA1c) significantly decreases compared to those treated with multiple daily insulin injections, and the total insulin dosage may also be reduced. Intensive therapy can rapidly lower blood glucose levels, alleviate hyperglycemic toxicity, and improve insulin resistance and pancreatic β‐cell function [10]. However, the impact of CSII on the Cardiometabolic Index has not yet been reported in relevant studies. Therefore, this study aims to evaluate the effect of CSII on cardiac metabolism in newly diagnosed T2DM subjects by observing changes in CMI values before and after treatment, and further explore the correlation between CMI and insulin resistance.

## 2 Methods

### 2.1 Study Population

A retrospective collection was conducted on 604 patients who were hospitalised in the Department of Endocrinology at the Affiliated Hospital of Jiangsu University. According to the 1999 WHO diagnostic criteria for T2DM, the blood glucose inclusion criteria for this study are: typical symptoms of diabetes plus fasting plasma glucose (FPG) ≥ 7.0 mmol/L, or random blood glucose ≥ 11.1 mmol/L, or 2‐h post‐OGTT plasma glucose (2hPG) ≥ 11.1 mmol/L. For individuals without typical symptoms of diabetes, blood glucose re‐testing is required for confirmation. The study included newly diagnosed patients with type 2 diabetes mellitus (T2DM) who met the following glycemic inclusion criteria: glycated haemoglobin A1c (HbA1c) level ≥ 9.0%, or fasting plasma glucose (FPG) ≥ 11.1 mmol/L; and who agreed to receive CSII therapy. Among them, there were 408 males (67.5%) and 196 females (32.5%). The mean age of the patients was 50.64 ± 12.24 years.

Exclusion criteria were as follows: (1) Type 1 diabetes mellitus, gestational diabetes mellitus, and specific types of diabetes (e.g., monogenic diabetes); (2) Acute complications and Chronic complication (Acute complications include diabetic ketoacidosis, hyperosmolar nonketotic diabetic coma, and lactic acidosis. Chronic complications include diabetic cardiovascular complications (with a history of coronary heart disease, cerebrovascular disease, or peripheral arterial disease), diabetic nephropathy, diabetic foot, and diabetic peripheral neuropathy); (3) Organ dysfunction, including liver failure, heart failure, respiratory failure, renal failure, etc.; (4) Coexisting non‐diabetic macrovascular diseases with a definitive diagnosis, such as coronary heart disease, cerebrovascular disease, and peripheral arterial sclerosis; malignancies; a history of major surgery or trauma within the past 6 months; autoimmune diseases (e.g., systemic lupus erythematosus, rheumatoid arthritis); uncontrolled hyperthyroidism or hypothyroidism; adrenal diseases (Cushing's syndrome, pheochromocytoma, hyperaldosteronism, etc.); a history of infection or antibiotic use within the past 2 weeks; (5) Other exclusion factors: long‐term bedridden status, mental disorders (Alzheimer's disease) that render the patient unable to cooperate with the study; (6) Previous use of hypoglycemic medications.

### 2.2 Research Methods

Basic information and general clinical data of the subjects were collected, along with results from glucose tolerance tests, insulin and C‐peptide stimulation tests, and blood biochemical analyses. Fasting plasma glucose (FPG) and 2‐h postprandial plasma glucose (2hPPG) were measured using the glucose oxidase‐peroxidase colorimetric method. The concentrations of fasting insulin (FIns), 2‐h postprandial insulin (2hINS), fasting C‐peptide (FCP), and 2‐h postprandial C‐peptide (2hCP) were determined by chemiluminescent immunoassay. The level of glycated haemoglobin A1c (HbA1c) was detected using high‐performance liquid chromatography. Biochemical indicators related to lipid metabolism, including TG, total cholesterol (TC), HDL‐C, and low‐density lipoprotein cholesterol (LDL‐C), were measured using an automatic biochemical analyser (Au2700).

Calculation of Indices:

$$\text{Body mass index}\;\left(\mathrm{BMI}\right)=\text{Weight}\;\left(\mathrm{kg}\right)/{\left[\text{Height}\;\left(m\right)\right]}^{2}$$

$$\text{HOMA}-\mathrm{IR}=\mathrm{FPG}\;\left(\text{mmol}/L\right)\times \text{FIns}\;\left(\mathrm{\mu U}/\mathrm{mL}\right)/22.5$$

$$\text{HOMA}-\beta =20\times \left[\text{FIns}\;\left(\mathrm{\mu U}/\mathrm{mL}\right)/\left(\mathrm{FPG}\;\left(\text{mmol}/L\right)-3.5\right)\right]$$

$$\mathrm{CMI}=\left(\text{Waist circumference}/\text{Height}\right)/\left(\mathrm{TG}/\mathrm{HDL}-C\right)$$

### 2.3 CSII Treatment Protocol

For newly diagnosed patients with T2DM, the starting doses of insulin pumps vary, calculated based on the total daily dose (U) = body weight (kg) × 0.5–0.6 U/kg. The basal infusion rate accounts for 40%–60% of the total daily insulin, while the pre‐meal bolus doses are evenly distributed as one‐third each for breakfast, lunch, and dinner. During CSII therapy, finger‐prick blood glucose levels are monitored seven times daily, including before and after each of the three meals, as well as at bedtime. Adjust the insulin dosage in a timely manner based on blood glucose levels. During the treatment period, patients should adhere to dietary and exercise therapy. The time interval between discontinuation of the CSII and insulin level measurement ranges from 24 to 48 h.

### 2.4 Statistical Methods

Statistical analysis was performed using SPSS 25.0. A *p*‐value of less than 0.05 was considered statistically significant. Quantitative data underwent a normality test. For measurement data that did not follow a normal distribution, the median (interquartile range) was used for representation. When the data did not conform to a normal distribution, non‐parametric tests (Mann–Whitney *U* test) were employed for inter‐group comparisons. The correlation between the CMI and various variables was assessed using the Spearman correlation test. After normalizing non‐normally distributed data through appropriate transformations, multivariate stepwise linear regression analysis was conducted.

## 3 Results

### 3.1 Comparison of CMI and Metabolic Indicators Before and After CSII Therapy

A comparison of the changes in metabolic indicators before and after insulin pump therapy revealed that the level of the CMI significantly decreased post‐treatment. In terms of glucose metabolism, FPG levels markedly declined after treatment, and 2hPPG levels also showed a significant reduction. Regarding pancreatic islet function, the HOMA‐IR decreased post‐treatment, while the HOMA‐β significantly increased, indicating an improvement in insulin resistance and an enhancement of pancreatic β‐cell function. In terms of lipid metabolism, TG, LDL‐C, and TC all significantly decreased after treatment, whereas HDL‐C also showed a slight decline. Regarding blood pressure, both systolic and diastolic blood pressure levels significantly decreased post‐treatment (Table 1).

**TABLE 1:** Comparison of various indicators before and after CSII therapy.

Variables	Pre‐treatment	Post‐treatment	*Z*	*p*
Age, year	50.64 ± 12.24	—	—	—
Gender (female), *n* (%)	196 (32.5%)	—	—	—
CMI	0.73 (0.43, 1.42)	0.64 (0.39, 1.17)	−3.071	0.002
HOMA‐IR	2.60 (1.71, 4.00)	1.30 (0.69, 1.86)	−18.991	< 0.001
HOMA‐β	12.30 (6.98, 20.92)	23.79 (12.74, 37.93)	−12.270	< 0.001
BMI (kg/m^2^)	24.97 (22.85, 27.30)	24.24 (22.41, 26.53)	−3.403	0.001
SBP (mmHg)	130 (120, 140)	120 (119, 130)	−10.323	< 0.001
DBP (mmHg)	80 (76, 90)	75 (70, 80)	−11.536	< 0.001
HbA1c (%)	10.24 ± 1.88	—	—	—
WC (cm)	89.1 (82.1, 95.43)	87 (81.7, 95)	2.351	0.019
FPG (mmol/L)	11.96 (9.94, 14.32)	6.92 (6.13, 7.80)	−27.451	< 0.001
2hPPG (mmol/L)	21.93 (18.93, 24.92)	13.52 (11.38, 15.79)	−26.182	< 0.001
FIns (μIU/L)	4.76 (3.31, 7.51)	4.34 (2.26, 6.10)	−6.554	< 0.001
2hIns (μIU/L)	15.41 (9.20, 28.37)	37.51 (17.21, 57.69)	−13.112	< 0.001
FCP (ng/mL)	2.10 (1.55, 2.78)	2.24 (1.67, 2.72)	−1.671	0.095
2hCP (ng/mL)	3.94 (2.84, 5.47)	6.18 (4.61, 8.29)	−14.495	< 0.001
TG (mmol/L)	2.28 (1.53, 3.65)	1.57 (1.18, 2.04)	−11.978	< 0.001
HDL‐C (mmol/L)	1.72 (1.15, 2.45)	1.35 (0.84, 2.05)	−7.637	< 0.001
LDL‐C (mmol/L)	3.04 (2.39, 3.77)	2.59 (2.15, 3.17)	−7.995	< 0.001
TC (mmol/L)	5.03 (4.39, 5.82)	3.81 (2.99, 4.73)	−16.916	< 0.001
Metformin (*n*)	—	183	—	—
DPP‐4 inhibitors (*n*)	—	93	—	—
α‐glucosidase inhibitors (*n*)	—	7	—	—
Lipid‐lowering agents (*n*)	—	63	—	—

Abbreviations: 2hIns, postprandial plasma insulin; 2hPPG, postprandial plasma glucose; DBP, diastolic blood pressure; FIns, fasting plasma insulin; FPG, fasting plasma glucose; HbA1c, glycosylated haemoglobin c; HDL‐C, high‐density lipoprotein cholesterol; HOMA‐IR, Homeostasis Model Assessment of Insulin Resistance; HOMA‐β, Homeostatic Model Assessment of β‐Cell Function; LDL‐C, low‐density lipoprotein cholesterol; SBP, systolic blood pressure; TC, total cholesterol; TG, triglyceride.

### 3.2 Correlation Analysis Between CMI and Various Indicators in Newly Diagnosed T2DM Patients

Table 2 presents the correlation analysis between the CMI and various indicators in newly diagnosed T2DM patients before CSII treatment. The results revealed that CMI was positively correlated with the HOMA‐IR, BMI, FIns, 2hIns, FCP, 2hCP, TG, TC. Conversely, CMI exhibited a negative correlation with HDL‐C、HOMA‐β (Table 2).

**TABLE 2:** Correlation analysis of CMI with various indicators before and after CSII treatment in T2DM.

Various	Pre‐treatment		Post‐treatment		Various	Difference before and after treatment	
	*r*	*p*	*r*	*p*		*r*	*p*
HOMA‐IR	0.24	< 0.001	0.166	< 0.001	ΔHOMA‐IR	0.205	< 0.001
HOMA‐β	−0.116	0.004	−0.108	0.008	ΔHOMA‐β	0.054	0.186
BMI	0.18	< 0.001	0.156	< 0.001	ΔBMI	−0.030	0.456
SBP	0.051	0.214	−0.004	0.921	ΔSBP	0.032	0.432
DBP	0.057	0.161	0.018	0.659	ΔDBP	0.070	0.084
FPG	0.061	0.134	0.054	0.184	ΔFPG	0.091	0.026
2hPPG	0.001	0.981	−0.02	0.626	Δ2hPG	0.044	0.285
FIns	0.202	< 0.001	0.163	< 0.001	ΔFIns	0.129	0.001
2hIns	0.108	0.008	0.01	0.799	Δ2hIns	0.012	0.761
FCP	0.311	< 0.001	0.222	< 0.001	ΔFCP	0.161	< 0.001
2hCP	0.181	< 0.001	0.125	0.002	Δ2hCP	−0.019	0.641
TG	0.811	< 0.001	0.552	< 0.001	ΔTG	0.836	< 0.001
HDL‐C	−0.642	< 0.001	−0.791	< 0.001	ΔHDL‐C	−0.166	< 0.001
LDL‐C	−0.018	0.654	−0.003	0.949	ΔLDL‐C	−0.041	0.317
TC	0.206	< 0.001	0.137	< 0.001	ΔTC	−0.014	0.734

The correlation analysis between CMI and various indicators in newly diagnosed T2DM patients after CSII treatment. The findings indicated that CMI remained positively correlated with HOMA‐IR, BMI, FIns, FCP, 2hCP, TG, and TC. Similarly, CMI continued to demonstrate a negative correlation with HDL‐C and HOMA‐β (Table 2).

The differences in various indicators before and after treatment are denoted by Δ. Correlation analysis revealed that ΔCMI was positively correlated with ΔHOMA‐IR, ΔFPG, ΔFIns, ΔFCP, and ΔTG, while negatively correlated with ΔHDL‐C (Table 2).

### 3.3 Stepwise Linear Regression Analysis of CMI and Various Indicators Before and After CSII Treatment

Using CMI before and after treatment as the dependent variable, and blood glucose, insulin, C‐peptide, HOMA‐IR, and HOMA‐β before and after treatment as independent variables respectively, a stepwise linear regression model analysis was conducted. The results showed that before treatment, CMI is independently associated with FCP and FPG (Table 3). After treatment, CMI is associated with FCP and HOMA‐IR (Table 4). The ΔCMI is correlated with ΔTG and ΔHDL‐C (Table 5).

**TABLE 3:** Stepwise linear regression analysis of CMI and various indicators in newly diagnosed T2DM patients before CSII treatment.

Various	*β*	SE	*t*	*p*	95% CI
	−0.490	0.080	−6.102	< 0.001	−0.648, −0.333
FCP	0.102	0.014	7.170	< 0.001	0.074, 0.130
FPG	0.014	0.006	2.532	0.012	0.003, 0.025

**TABLE 4:** Stepwise linear regression analysis of CMI and various indicators in newly diagnosed T2DM patients after CSII treatment.

Various	*β*	SE	*t*	*p*	95% CI
	−0.403	0.037	−10.971	< 0.001	−0.475, −0.331
FCP	0.062	0.012	5.355	< 0.001	0.039, 0.085
HOMA‐IR	0.065	0.018	3.706	< 0.001	0.031, 0.100

**TABLE 5:** Stepwise linear regression analysis of ΔCMI and various indicators in newly diagnosed T2DM patients after CSII treatment.

Various	*β*	SE	*t*	*p*	95% CI
	0.132	0.071	1.851	0.065	−0.008, 0.273
ΔTG	0.431	0.015	29.15	< 0.001	0.402, 0.46
ΔHDL‐C	−0.964	0.147	−6.547	< 0.001	−1.254, −0.675

## 4 Discussion

This study analysed the impact of short‐term CSII therapy on the CMI in patients with T2DM. It was found that CSII significantly improved glycemic control and insulin resistance while effectively reducing CMI levels. This result provides new evidence for understanding the metabolic and cardiovascular benefits of CSII in T2DM management.

Insulin resistance and impaired pancreatic β‐cell function are two crucial pathophysiological mechanisms underlying the onset and progression of T2DM. Studies have demonstrated that after CSII treatment, T2DM patients exhibit significant improvement in blood glucose levels, along with amelioration in the degree of insulin resistance and β‐cell function [11, 12]. The findings of this study reveal that after CSII therapy, patients showed a marked decrease in FPG, 2hPG, and HOMA‐IR levels, accompanied by a significant increase in the HOMA‐β. These results suggest that intensive glycemic control with CSII can, to a certain extent, improve insulin resistance and pancreatic β‐cell function.

As a comprehensive index integrating visceral fat distribution and dyslipidemia, the CMI has been proven in recent years to be closely associated with various metabolic and cardiovascular outcomes. Multiple cross‐sectional and cohort studies have demonstrated that an elevated CMI is significantly correlated with insulin resistance, impaired glucose tolerance, and an increased risk of T2DM [4, 5]. This study found a positive correlation between ΔCMI and ΔFPG. A clinical study utilising data from the National Health and Nutrition Examination Survey (NHANES) revealed a significant positive correlation between CMI and myocardial infarction risk, with restricted cubic spline (RCS) curves demonstrating a linear relationship between CMI and myocardial infarction [13]. Multivariable Cox proportional hazards analysis indicated that an elevated CMI was significantly associated with both all‐cause and cardiovascular mortality. Kaplan–Meier survival curves demonstrated significantly worse outcomes in terms of all‐cause and cardiovascular mortality among individuals with higher CMI levels. This cohort study also found a significant correlation between CMI and both all‐cause and cardiovascular mortality [14]. A notable positive correlation was also observed between CMI and heart failure. ROC curve analysis demonstrated that CMI had good diagnostic value for heart failure, outperforming traditional indicators such as BMI [15]. This study is the first to observe a significant decline in CMI levels in T2DM patients following short‐term CSII therapy, suggesting that this treatment may contribute to improving patients' cardiovascular metabolic health status.

This study further explored the underlying mechanisms through correlation and regression analyses. The results indicated a positive correlation between CMI and the HOMA‐IR, with HOMA‐IR and FCP being independent influencing factors of CMI. Changes in CMI were positively correlated with ΔHOMA‐IR, ΔFPG, ΔFIns, ΔFCP, and ΔTG, while negatively correlated with ΔHDL‐C. This suggests that the decline in CMI achieved by CSII therapy is likely partly attributable to its effects in improving insulin resistance and pancreatic islet function. Elevated CMI levels are closely associated with an increased risk of cardiovascular diseases (CVDs) and serve as an important potential biomarker for assessing cardiovascular risk [16]. CMI combines WHtR and TG/HDL‐C, reflecting central obesity and dyslipidemia, both of which are key factors contributing to elevated cardiovascular risk in diabetes. The TG/HDL‐C ratio, a crucial component of CMI, is characterised by elevated TG and reduced HDL‐C levels. High TG is a risk factor for CVDs [17, 18]. Glycolipoproteins, their remnants, and specific markers of TG metabolism, such as lipoprotein lipase (LPL) and apolipoprotein C‐III (apoC‐III), promote endothelial dysfunction, inflammation, and foam cell formation, directly or indirectly facilitating the progression of atherosclerosis and CVDs [19]. Another potential mechanism may involve the role of visceral fat accumulation in driving insulin resistance. Increased visceral fat leads to elevated production of pro‐inflammatory cytokines, such as interleukin‐6 (IL‐6) and tumour necrosis factor‐α (TNF‐α), which impair endothelial function and promote the development of atherosclerosis [20, 21]. Insulin resistance can increase cardiovascular risk through multiple pathways, such as promoting atherosclerosis and activating the sympathetic nervous and renin‐angiotensin systems [22, 23]. In this study, we found that after CSII treatment, CMI significantly decreased, accompanied by improved insulin resistance, and notably reduced blood glucose levels, as well as improved lipid profiles. These findings suggest that a reduction in CMI may lower the risk of CVDs by alleviating insulin resistance, reducing hyperglycemic and lipotoxic effects. Focusing on the high‐risk population of T2DM, this study revealed that CSII therapy could significantly reduce CMI and improve insulin resistance, blood glucose, and lipid levels, which are indicators related to cardiovascular risk. Clinically, monitoring changes in CMI can be used to evaluate the regulatory effect of intensive CSII glycemic control on cardiovascular risk, providing a reference for individualised adjustment of treatment plans, making treatment more targeted, and enriching the clinical management strategies for intensive CSII glycemic control.

The limitations in terms of geographical location and population may restrict the applicability of the study results to other populations or healthcare settings. In the future, multi‐center, large‐sample prospective studies are required to verify its universality and stability. Secondly, the observation period of this study was relatively short, mainly evaluating the metabolic changes within 14 days after CSII therapy. Although significant biochemical improvements were observed during this period, the dynamic trend of CMI during long‐term interventions and its long‐term correlation with the occurrence of complications have not been explored.

## 5 Conclusion

The results of this study indicate that the CMI levels significantly decreased in newly diagnosed patients with T2DM after CSII therapy. This suggests that short‐term insulin pump therapy for T2DM may play a certain role in improving cardiometabolic health. This study was a single‐center retrospective study, and all subjects were newly diagnosed T2DM patients from the same hospital.

## Author Contributions

X.D. designed the study. X.C. and S.L. contributed to conduct the research and collect data. Z.C., D.W. and L.Y. were responsible for analysing the data and writing the paper. X.D. and H.L. made suggestions and rev Affiliated Hospital of Jiangsu University Beigu Talent Cultivation Plan Project ised the paper. X.D. and H.L. made suggestions and revised the paper. The submitted version has been read and approved by all authors.

## Funding

This work was supported by the Projects from Social Development of Zhenjiang (SH2023029), the Affiliated Hospital of Jiangsu University Beigu Talent Cultivation Plan Project, the Elderly Health Research Project of Jiangsu Province (LR2021037).

## Ethics Statement

This study was approved by the Biomedical Research Ethics Committee of Jiangsu University Affiliated Hospital.

## Consent

The authors have nothing to report.

## Conflicts of Interest

The authors declare no conflicts of interest.

## Data Availability

The data supporting the results of this study is not publicly available due to potential harm to the privacy of study participants, but may be obtained from the corresponding author upon reasonable request (dengxia11@outlook.com) Obtained from here.
